# Supplementary material for: Polymer Membranes Sonocoated and Electrosprayed with Nano-Hydroxyapatite for Periodontal Tissues Regeneration
Source: Nanomaterials (Basel). 2019 Nov 15;9(11):1625. doi: 10.3390/nano9111625 (PMC6915502; doi:10.3390/nano9111625)
Supplement: Supplementary file 1 [file nanomaterials-09-01625-s001.pdf]

nanomaterials

Supplementary Materials

Polymer Membranes Sonocoated and Electrosprayed with Nano-Hydroxyapatite for Periodontal Tissues Regeneration

Julia Higuchi [1,2,3,*], Giuseppino Fortunato [3], Bartosz Woźniak [1], Agnieszka Chodara [1,2], Sebastian Domaschke [4,5], Sylwia Męczyńska-Wielgosz [6], Marcin Kruszewski [7], Alex Dommann [8] and Witold Łojkowski [1]**

[1] Laboratory of Nanostructures, Institute of High Pressure Physics, Polish Academy of Sciences, 01142 Warsaw, Poland; b.wozniak@labnano.pl; a.chodara@labnano.pl; w.lojkowski@labnano.pl
[2] Faculty of Materials Science and Engineering, Warsaw University of Technology, 02507 Warsaw, Poland
[3] Laboratory for Biomimetic Membranes and Textiles, Empa Swiss Federal Laboratories for Materials Science and Technology, 9014 St. Gallen, Switzerland;
[4] Experimental Continuum Mechanics, Empa Swiss Federal Laboratories for Materials Science and Technology, 8600 Dübendorf, Switzerland; sebastian.domaschke@empa.ch
[5] Department of Mechanical and Process Engineering, Institute for Mechanical Systems, ETH Zürich, 8092 Zürich, Switzerland
[6] Centre for Radiobiology and Biological Dosimetry, Institute of Nuclear Chemistry and Technology, 03195 Warsaw, Poland; sylwia.meczynska@gmail.com
[7] Department of Molecular Biology and Translational Research, Institute of Rural Health, 20090 Lublin, Poland; m.kruszewski@ichtj.waw.pl
[8] Department Materials meet Life, Empa Swiss Federal Laboratories for Materials Science and Technology, 9014 St. Gallen, Switzerland; Alex.Dommann@empa.ch

*Correspondence: j.higuchi@labnano.pl; Tel.: +48 22 876 0429
**Correspondence: Giuseppino.Fortunato@empa.ch; Tel: +41 58 765 7677

Table S1. Surface elemental composition (% atomic concentration) determined by XPS

	**Surface Elemental Composition [%]**			
**Sample**	**C**	**O**	**Ca**	**P**
PLGA	57,09	42,91	----	----
PDLLA	62,63	37,37	----	----
PDLLA/PLGA	60,91	39,09	----	----
PLGA/nHA	49,46	45,59	3,02	1,92
PDLLA/nHA	43,43	45,89	6,45	4,22
PDLLA/PLGA/nHA	47,32	44,45	4,91	3,32
